# Performance of the Transport Layer Security Handshake Over 6TiSCH

**DOI:** 10.3390/s21062192

**Published:** 2021-03-21

**Authors:** Timothy Claeys, Mališa Vučinić, Thomas Watteyne, Franck Rousseau, Bernard Tourancheau

**Affiliations:** 1 Inria, 2 Rue Simone IFF, 75012 Paris, France; malisa.vucinic@inria.fr (M.V.); thomas.watteyne@inria.fr (T.W.); 2 Univ. Grenoble Alpes, CNRS, Grenoble INP, LIG, F-38000 Grenoble, France; franck.rousseau@univ-grenoble-alpes.fr (F.R.); bernard.tourancheau@univ-grenoble-alpes.fr (B.T.)

**Keywords:** internet of things, TLS, DTLS, 6TiSCH, TSCH, OpenWSN

## Abstract

This paper presents a thorough comparison of the Transport Layer Security (TLS) v1.2 and Datagram TLS (DTLS) v1.2 handshake in 6TiSCH networks. TLS and DTLS play a crucial role in protecting daily Internet traffic, while 6TiSCH is a major low-power link layer technology for the IoT. In recent years, DTLS has been the de-facto security protocol to protect IoT application traffic, mainly because it runs over lightweight, unreliable transport protocols, i.e., UDP. However, unlike the DTLS record layer, the handshake requires reliable message delivery. It, therefore, incorporates sequence numbers, a retransmission timer, and a fragmentation algorithm. Our goal is to study how well these mechanisms perform, in the constrained setting of 6TiSCH, compared to TCP’s reliability algorithms, relied upon by TLS. We port the mbedTLS library to OpenWSN, a 6TiSCH reference implementation, and deploy the code on the state-of-the-art OpenMote platform. We show that, when the peers use an ideal channel, the DTLS handshake uses up to 800 less and completes 0.6 s faster. Nonetheless, using an unreliable communication link, the DTLS handshake duration suffers a performance penalty of roughly 45%, while TLS’ handshake duration degrades by merely 15%. Similarly, the number of exchanged bytes doubles for DTLS while for TLS the increase is limited to 15%. The results indicate that IoT product developers should account for network characteristics when selecting a security protocol. Neglecting to do so can negatively impact the battery lifetime of the entire constrained network.

## 1. Introduction

With the Internet of Things (IoT) networking stack fully standardized and operational [1], the research community and industry now face the daunting task of designing and deploying network security protocols. The low-power nature and limited computational capabilities of many IoT devices make the design and integration of these protocols challenging. IoT product developers have several established and novel security schemes at their disposal to protect application traffic. On the one hand, there is a new suite of object security protocols that aim for low-power consumption and optimized encoding schemes [2,3]. On the other hand, developers can use the well-known transport layer security protocols: Transport Layer Security (TLS) [4] and Datagram Transport Layer Security (DTLS) [5]. Both TLS and DTLS are mature protocols and enjoy extensive support. For IoT deployments, they are available through state-of-the-art libraries such as mbedTLS [6] and wolfSSL [7]. These libraries are tailored for constrained environments and provide open-source, vetted implementations.

In contrast to TLS, DTLS can run over unreliable transport such as UDP. Consequently, it has been the preferred choice for protecting IoT application traffic, as reliable transport such as TCP has a non-negligible overhead. In addition to the larger header, the TCP acknowledgment packets consume much of the already scarce bandwidth in constrained networks. Although the DTLS record protocol tolerates packet loss, the handshake does require reliable message delivery. The handshake performs a key exchange to establish a shared secret between two peers. The record protocol then uses the secret to encrypt application traffic. The handshake is arguably the most demanding phase of the DTLS protocol because of the potentially large message sizes and its reliance on asymmetric cryptography. To obtain reliability during the handshake phase, DTLS implements multiple TCP-like features: it adds sequence numbers to the DTLS headers and employs a naive retransmission mechanism with back-off. DTLS also supports message fragmentation during the handshake to prevent oversized UDP datagrams that would otherwise result in IP fragmentation and potential packet loss.

In this article, we compare the network performance of the TLSv1.2 and DTLSv1.2 handshake in constrained environments. We undertake an experimental study of both handshake protocols when the messages travel over a multihop 6TiSCH network. The 6TiSCH specification is a core technology of the IoT. It extends the TSCH (Time-Slotted Channel Hopping) MAC layer specified in the IEEE802.15.4 amendment [8] to be IP-compliant. Originally designed for industrial networks, TSCH has proven to yield robust and reliable end-to-end communication in a plethora of harsh environments [9] while still minimizing energy consumption. We quantify how the reliability mechanisms in the DTLS handshake impact its performance in a 6TiSCH network, compared to the TLS handshake. We show that changes to the handshake message size or retransmission policy have a non-negligible impact on the operation of the 6TiSCH network. In the first part of this work, we ported the mbedTLS library on top of OpenWSN [10], an open-source reference implementation of 6TiSCH. We run our code on the OpenMote platform, an open hardware initiative designed for low-power IEEE802.15.4 networks. We show that the reliability of the underlying communication link significantly impacts the performance of the TLS and DTLS handshake. In scenarios with 100% end-to-end reliability, the performance of the DTLS handshake is slightly better. It transmits between 500 and 800 less and completes approximately 0.6 s faster. However, when the end-to-end reliability deteriorates, the performance of DTLS degrades rapidly. If a transport layer packet has a 5% probability of getting lost, the median handshake duration for DTLS more than doubles, and it uses from ±37% to ±45% more bytes. TLS performance remains more stable. The handshake duration grows between ±10% and ±15%, and the number of exchanged bytes grows between ±2% and ±15%.

In the second part of the paper, we provide an insightful discussion on the results. We detail the different mechanisms at work which cause the performance discrepancy and explain how we can use the many TCP options to limit the TLS handshake and TCP overhead in constrained environments.

Finally, we propose an improvement for the TLS handshake. We make use of a Performance Enhancing Proxy (PEP) [11] at the 6TiSCH network gateway to reduce the spurious retransmissions incurred by the high latency in 6TiSCH networks. The PEP reduces the number of exchanged bytes during the TLS handshake and the total duration of the TLS handshake.

The remainder of this article is organized as follows. Section 2 provides the relevant background on 6TiSCH and the (D)TLS handshake protocol and lists the important differences between the TLS and DTLS handshake. Section 3 presents the experiments and results. Section 4 describes the different algorithms and mechanisms at work throughout the network stack to successfully complete the handshake, and identifies the root causes for the results obtained in Section 3. Based on the insights from the previous section, Section 5 implements and evaluates the PEP setup for the TLS handshake. Section 6 sheds some light on the changes in recently standardized TLSv1.3 and upcoming DTLSv1.3 specifications. Section 7 discusses related works. Finally, Section 8 concludes this article and presents avenues for future work.

## 2. Background

### 2.1. TSCH, 6TiSCH, and IPv6

**Figure 1:**
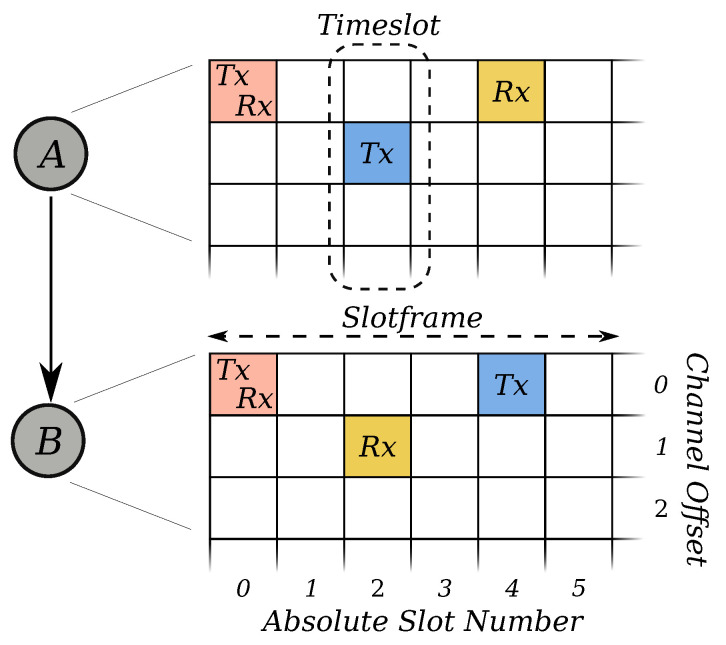
A typical TSCH schedule shared between device A and B, containing a Tx, an Rx, and a TxRx slot for communication.

The IEEE802.15.4e amendment [12] first standardized the TSCH mode. It uses a sparse time-slotted schedule, depicted in Figure 1, combined with channel hopping over 16 distinct frequencies to provide robust communication in multihop networks. Each TSCH device maintains its schedule. The schedule synchronizes the device’s access to the wireless transmission medium and prevents collisions. Channel hopping provides frequency diversity in the crowded 2.4 GHz ISM band. The schedule consists of a repeating structure, called “slotframe”. A slotframe, in turn, consists of a group of cells. The individual cells describe when the device should wake up to communicate with its neighbors and when it should sleep to save battery. There are three active cell types: transmission slots (Tx), reception slots (Rx), and  shared slots (TxRx). A single cell in the schedule is long enough to send a maximum length IEEE802.15.4 frame ( 127 ) and receive a short acknowledgment frame. While the exact duration of a cell is implementation-specific, 10 ms, 15 ms, and 20 ms are commonly used.

To support dynamic traffic patterns on top of the TSCH schedule, the 6TiSCH Working Group (WG) designed the 6TiSCH Operation Sublayer (6top) Protocol (6P) [13]. 6P is a pairwise negotiation protocol that enables TSCH neighbors to allocate and delete cells in their schedules [14]. The protocol defines seven commands: *ADD*, *DELETE*, *RELOCATE*, *COUNT*, *LIST*, *SIGNAL*, and  *CLEAR*. A device uses these commands to manipulate its neighbor’s schedule and requests an increase or decrease of the available bandwidth. The Scheduling Function (SF), a separate module of the 6TiSCH specification, uses the 6P protocol to implement a specific TSCH cell allocation policy. The policy depends on the traffic patterns of the upper-layer protocols and applications. The 6TiSCH WG defined a default scheduling policy called Minimal Scheduling Function (MSF). MSF identifies two types of cells in the schedule: autonomous cells and managed cells. Nodes install autonomous cells to provide minimal bandwidth to their neighbors and managed cells to respond dynamically to a varying traffic load in the network.

6TiSCH networks are IPv6 compliant thanks to the IETF 6LoWPAN adaptation layer. The 6LoWPAN layer optimizes the limited IEEE802.15.4 payload space by compressing the headers of upper layers. It also defines mechanisms for the support of operations required in IPv6, such as neighbor discovery and address autoconfiguration. Since IPv6-compliant networks must be capable of handling IP packets with a payload of at least 1280  [15], 6LoWPAN specifies a fragmentation mechanism. The algorithm iterates over the IPv6 packet and slices it into fragments according to the maximum frame size at the link layer, 127 B for IEEE802.15.4.

### 2.2. Applicability of TCP in the IoT

The main current transport layer protocols in IP-based IoT scenarios are UDP and TCP. Due to TCP’s complexity, header size, acknowledgment packet overhead, and unsuitability for multicast traffic, many IoT deployments prefer UDP. However, the basic TCP protocol is extendable with many options. Through careful selection of several TCP options, an implementation can become more lightweight [16]. The Lightweight Implementation Guide (LWIG) group at the IETF defined a set of TCP options to improve TCP’s performance in constrained networks [16]. Here, we briefly describe the options we use during our performance evaluation.

#### 2.2.1. Maximum Segment Size

During the TCP handshake a host can announce the maximum TCP segment size it can accept. The host can arbitrarily choose the value of the Maximum Segment Size (MSS). The option does not incur any additional overhead in the TCP header. Smaller segment sizes are useful when constrained devices only have limited memory resources. Long segments require not only big TCP send and receive buffers but also increase the 6LoWPAN fragmentation rate. Constrained devices need to allocate additional buffers to store the 6LoWPAN fragments; the loss of a single fragment causes a retransmission of the entire TCP segment.

#### 2.2.2. Selective Acknowledgments

Selective Acknowledgments (SACK) allow the receiving peer to precisely indicate in its acknowledgment packet which TCP segments were not received. A single SACK block adds 10 to an acknowledgment packet, and it specifies the TCP sequence numbers of the missing data. This option is only useful when the sender has multiple unacknowledged segments in flight. Upon reception of a SACK, the sender creates a new TCP segment that contains only the data the receiver has requested.

#### 2.2.3. Delayed Acknowledgements

Default TCP operation requires a corresponding acknowledgment for every received segment. Delayed acknowledgments try to reduce the number of acknowledgment packets on the wire. In a stream of full-sized segments, the receiver can delay the transmission of an acknowledgment. It can wait for the next segment to arrive. The delayed acknowledgment then acknowledges both segments cumulatively. There should be an acknowledgment for at least every second segment. In addition, the delay should not extend more than 0.5 s [17].

#### 2.2.4. Nagle’s Algorithm

Nagle’s algorithm [18], shown in Algorithm 1, limits the amount of data in flight to a single full-sized segment (=MSS) unless another full-sized segment is available for transmission.

**Algorithm 1** Nagle’s Algorithm
1:**procedure**Transmit(*D*)2:    **if** window size >= MSS and |D|>= MSS **then**3:        Send(D)4:    **else**5:        **if** data in flight **then**6:           Queue(D)7:        **else**8:           Send(D)


Constrained devices typically use statically-allocated packet buffers. If the MSS value corresponds to the internal packet buffer size, Nagle’s algorithm ensures that the buffers are utilized at maximum efficiency. Without Nagle, many small segments could occupy buffer spaces foreseen for larger TCP segments, thereby wasting RAM.

### 2.3. The (D)TLS Handshake Protocol

Both TLSv1.2 and DTLSv1.2 are hybrid cryptosystems. They combine symmetric-key cryptography (for bulk encryption of application traffic) and public-key cryptography (to establish keys for the symmetric algorithms) in a single protocol. They consist of four subprotocols: the record protocol, the handshake protocol, the change cipher specification, and  the alert protocol. The record layer encapsulates the three other subprotocols. The RFCs specifying (D)TLSv1.2 [4,5] also use the term fragment to refer to the body of a record layer message. To avoid confusion with messages that are the explicit result of a fragmentation mechanism—possibly on other layers of the networking stack—in this article we use the term record fragment to denote the body of a record layer message.

#### Handshake Flow

**Figure 2:**
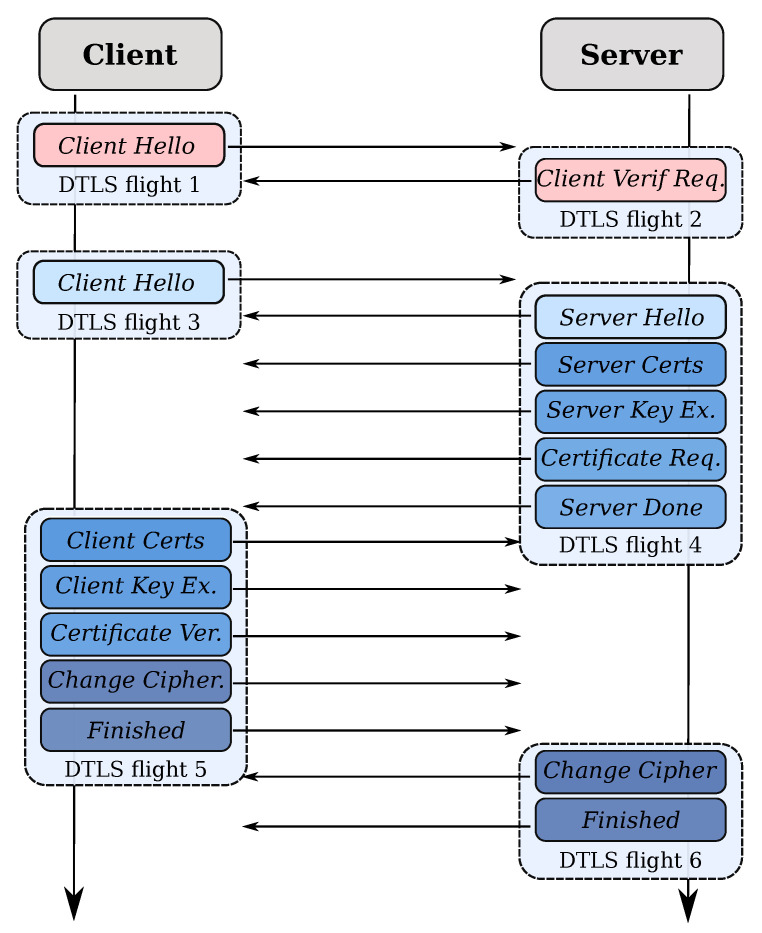
The (D)TLS handshake protocol. The initial two messages are only available when DTLS enforces Denial-of-Service protection.

The handshake protocol is responsible for negotiating a session that contains a session identifier, an optional peer certificate (X.509v3), a compression method, a cipher suite, a master secret, and  a boolean value stating if the session is resumable. The handshake happens in different phases. We illustrated the message flow in Figure 2.

Initially, the peers exchange hello messages (*ClientHello* and *ServerHello*) to agree on a compression method and cryptographic algorithms. The messages also contain randomness and state the support for session resumption. The client can request additional functionalities by including extensions in its hello message.

In the second phase, the peers transfer cryptographic parameters to derive the shared master secret. They also swap credential information to perform (mutual) authentication. Depending on the authentication method and the selected cipher suite, for example, when they use ECDHE_ECDSA as a key exchange method, the server sends a *ServerCertificate* message. This message contains a list of X.509v3 certificates. The first certificate in the chain is the server’s certificate, the last one is a self-signed certificate, potentially from a root Certificate Authority (CA), and represents the trust anchor. TLS and DTLS also support Pre-shared key (PSK) and raw public key (RPK) authentication. Following the server’s certificate chain and if the client and the server negotiated an ephemeral key exchange mechanism, the server sends a *ServerKeyExchange* message. In the case of a static key exchange algorithm, the client already has all the information necessary to continue with the key establishment. When the server wishes to perform mutual authentication, the server requests a client certificate through a *CertificateRequest* message. The server then notifies the client with the *ServerDone* message that it is done sending. In response to a *CertificateRequest* message, the client immediately sends its certificate chain. The client then follows up with the *ClientKeyExchange* and the *CertificateVerify* messages. The last two messages provide the server with the client’s key share and a signature calculated with the client’s private key over the message transcript. The server can verify the authenticity of the client and derive the shared master secret.

In the final phase of the handshake protocol, the client sends a *ChangeCipherSpec* message, indicating that the newly derived session key was installed. This message must arrive before the client’s last message, the *Finished message*. The latter contains the shared master secret, a string, and a hash of the entire handshake encrypted with the negotiated encryption algorithm and the new session key. The server responds with its *ChangeCipherSpec* message and *Finished* message. The final round-trip allows both peers to verify all handshake steps were successful.

### 2.4. Differences between TLS and DTLS

TLS and DTLS follow the same protocol flow as depicted in Figure 2. However, there are some significant adjustments to DTLS due to the unreliability of the UDP transport layer.

#### 2.4.1. Record Layer Changes

The DTLS record layer header has two additional fields compared to TLS. An epoch field and a sequence number field called the Record Sequence Number (RSN). Endpoints use epoch numbers to determine which cipher suite protected the record fragment. The endpoints increment the epoch numbers on each *ChangeCipherSpec* message. Epoch numbers resolve the ambiguity situation when data loss occurs during a session renegotiation or when multiple handshakes happen in close succession. TLS employs implicit sequence numbers for replay protection. The Message Authentication Code (MAC) over the TLS records also incorporates the sequence numbers. RSNs play a similar role in DTLS but are explicitly specified since records can get lost or delivered out-of-order. The DTLS record layer combines the RSN and the epoch number in a single 64-bit value while computing the MAC. DTLS increments the RSNs for each record and resets them to zero whenever the cipher state rolls over due to a session renegotiation. Implementations must therefore make sure the RSN/epoch pair is unique. DTLS can optionally perform replay detection by using the sliding window mechanism (defined in RFC 2401 [19]).

DTLS records must fit into a single UDP datagram to prevent buffering of incomplete records on the DTLS record layer [20]. At the same size, DTLS records should not trigger IP fragmentation along the way. Loss of a single IP fragment would result in the loss of the entire datagram. In addition, Network Address Translation (NAT) devices and firewalls might drop IP fragments; IPv6 does no longer supports IP fragmentation by default. The DTLS specification [5] states that it is the responsibility of the application to perform Path Maximum Transmission Unit (PMTU) discovery, but caution is advised due to the following reasons: (1) the DTLS record framing expands the datagram size thereby lowering the effective PMTU from the application’s perspective, (2) DTLS handshake message can easily exceed the MTU. TLS does not suffer from these limitations since TCP treats the TLS records as a byte stream and can thus create arbitrarily-sized segments (potentially limited by the MSS option).

It is noteworthy that both TLS and DTLS support an extension that can explicitly limit the size of the record fragments, called Maximum Fragment Length (MFL) extension. When negotiated, MFL limits the size of the record fragments to either 512 B, 1024 B, 2048 B, or 4096 B. It is, however, not widely support by TLS servers.

#### 2.4.2. Handshake Protocol Changes

The unreliability of the transport layer had a major impact on the design of the DTLS handshake protocol. Unlike application data, the handshake protocol must exchange its messages reliably to derive a shared security context successfully. Therefore, the DTLS handshake protocol implements some of the mechanisms we can find in TCP. The handshake protocol uses a retransmission mechanism and message sequence numbers. Note that the handshake message sequence numbers are independent of the record layer sequence numbers.

**Figure 3:**
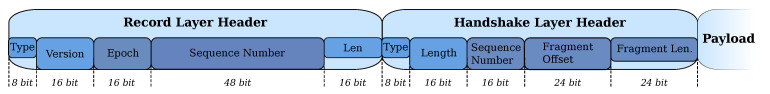
Illustration of the DTLS headers for a handshake message. In the record layer header, DTLS adds the epoch and sequence number fields. In the handshake header, DTLS adds sequence numbers, a fragment offset, and fragment length.

The retransmission operation works as follows. The DTLS handshake is divided into multiple flights, see Figure 2. Each time one of the endpoints sends an entire flight, it arms a timer. If the timer expires before the endpoint has received an answer, it resends the full previous flight. Since neither UDP nor DTLS uses acknowledgment packets, it not possible to inform the endpoint which individual DTLS messages inside the flight were received well and which ones got lost. Therefore, DTLS retransmits the entire flight. The default start timeout value is 1 s; it doubles each time the transmission fails, maxing out at 60 s. Handshake messages can grow larger than the Path Maximum Transmission Unit (PMTU) mostly due to long certificate chains. To prevent IP fragmentation, the DTLS handshake layer supports fragmentation. The handshake header has a fragment offset field and fragment length field to perform reassembly at the receiver side, see Figure 3.

DTLS uses a connectionless transport protocol, which makes it vulnerable to two types of Denial-of-Service (DoS) attacks. The first attack is a resource consumption attack. A large number of malicious clients send *ClientHello* packets to exhaust the resources of the server. The second attack is an amplification attack. Malicious clients spoof the IP address of a victim device and send *ClientHello* messages to the server. The server then responds with the next DTLS flight (containing the *ServerHello*, *ServerCertificate*, *ServerKeyExchange*, and *ServerDone* messages), potentially overwhelming the victim device. To mitigate these attacks, DTLS optionally uses a cookie exchange technique. At the start of the handshake protocol, the client must replay a cookie provided by the server to demonstrate that it can receive packets at its claimed IP address. The cookie exchange adds one full Round-Trip Time (RTT) to the DTLS handshake latency compared to TLS. Figure 2 depicts the technique in the first two messages. TLS does not suffer from the described attacks since the TCP handshake occurs before the client sends the first TLS message. It automatically detects address spoofing.

## 3. Handshake Performance Measurements

### 3.1. Setup

**Figure 4:**
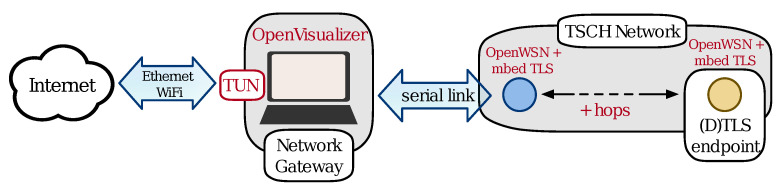
Experimental setup.

We consider the setting where a constrained 6TiSCH device, acting as a (D)TLS client, connects to a powerful (D)TLS server, see Figure 4. The constrained device uses the OpenWSN stack [10]; the powerful host uses the out-of-the-box Linux networking stack. The TCP/IP network stack of the server is unmodified and is unaware it is communicating with a constrained device. The (D)TLS handshake always uses mutual authentication through the exchange of certificates. The certificate chains contain only one X.509 certificate. The received certificate matches with a stored root certificate, acting as a trust anchor for authentication.

To obtain experimental results on the performance of the (D)TLS handshake on top of 6TiSCH, we port mbedTLS to the OpenWSN project. We implemented 6LoWPAN fragmentation and the TCP protocol for the OpenWSN stack. We designed two abstraction layers, called opendtls and opentls, which function as wrappers around the mbedTLS library and allow OpenWSN applications to trigger the handshake protocol.

We perform several experiments using different sets of configuration parameters for the 6TiSCH network and (D)TLS stack to assess the impact on the handshake performance. We principally evaluate two performance characteristics: the number of exchanged bytes—measured at the physical layer—and the handshake duration. Unless noted otherwise, we use the configuration depicted in Table 1.

We use the OpenMote hardware platform to perform our experiments. The OpenMote features the CC2538 SoC [21], which has 32 KiB of SRAM and a 32 MHz ARM Cortex-M3 processor. Additionally, it provides a cryptographic coprocessor for AES and SHA functions and an acceleration engine for several big integer and elliptic curve operations. Figure 4 shows the experimental setup. The root node of the 6TiSCH network connects through a serial interface to a PC implementing the network gateway.

The OpenWSN project also provides the gateway software, called OpenVisualizer, to connect the mesh network to the Internet. It implements 6LoWPAN compression, decompression, fragmentation, and reassembly of packets. It uses a tun interface to inject packets into the kernel of the PC and route them to their final destination.

### 3.2. Network Stack Configuration

#### 3.2.1. Maximum Transmission Unit and Handshake Message Size

We start investigating the impact of the handshake message size. Sizeable handshake messages lead to large IPv6 packets which are then fragmented in several 6LoWPAN fragments. Both the TLS/TCP and DTLS/UDP stack can set an upper bound for the handshake message size by activating the MFL extension. TLS can also use the MSS option provided by TCP to limit the TCP segment size. IPv6-compliant IoT devices should support an MTU of 1280 [15]. However, the tight memory constraints on low-power IoT devices make this a challenging requirement. For the OpenMote platform, the combined RAM usage of OpenWSN, TCP, and mbedTLS only allows IPv6 packets with a maximum payload size of 864 B. When we use UDP instead of TCP, the software stack can support IPv6 packets with a maximum payload of 1377 B. Table 2 shows the handshake duration and the total number of exchanged bytes between both endpoints to establish a secure connection. We count all the incoming and outgoing bytes on the physical layer that are part of the handshake, i.e., IEEE802.15.4 frames containing handshake data and TCP control packets.

Comparing the TLS handshake with the IPv6 MTUs at 864 bytes and 356 bytes indicates that larger MTU values result in fewer transmitted bytes. When more TLS data fits in a TCP segment, it reduces the overhead of the TCP header. Besides, there are fewer TCP acknowledgments necessary. Activating Nagle’s algorithm further reduces the header overhead and thus the number of transmitted bytes.

A DTLS client can use the MFL extension to inform the server of the maximum record fragment size it supports. With an IPv6 MTU of 1377 B, the node can use two MFL sizes: 512 B or 1024 B. The MFL sizes indicate the size of the record fragment. To derive the IPv6 payload size, we add 13 B for the DTLS record header and 4 B for the 6LoWPAN compressed UDP header. Similarly to TCP, a higher IPv6 MTU results in fewer datagrams to complete the handshake and less overhead caused by headers. Compared to TLS, DTLS uses fewer bytes to complete the handshake. Several factors contribute to this difference. UDP does not use acknowledgment packets, and UDP has a significantly smaller header size. The UDP header is even further compressed from the standard 8 B down to 4 B through 6LoWPAN header compression, while TCP uses an uncompressed header of 20 B.

A varying maximum handshake message size also influences the handshake duration. To time the duration of TLS, we start the clock when the client sends its *syn* segment to open the TCP connection. While timing the DTLS handshake latency, we start the clock when the client sends the initial ClientHello, triggering DoS protection on the server endpoint. To prevent spurious DTLS retransmissions, we set the DTLS timeout value to a conservative 3 s. We observe that the MTU only slightly impacts the overall duration of both the TLS and DTLS handshake. When we activate Nagle’s algorithm in TCP, it incurs an additional delay. Without Nagle, TCP can pipeline the segments, having multiple unacknowledged segments in transit. Nagle’s algorithm minimizes TCP header overhead but limits the number of unacknowledged segments in flight.

#### 3.2.2. TSCH Schedule

**Figure 5:**
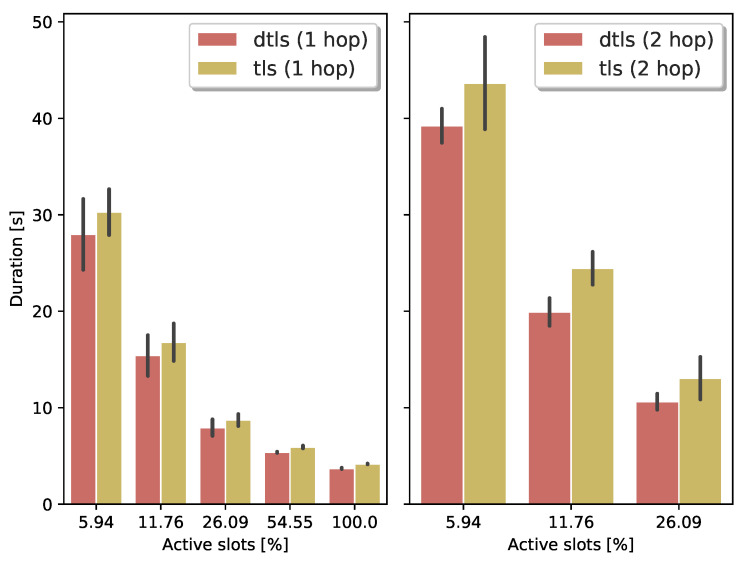
The duration for the (D)TLS handshake increases fast when the TSCH schedule is sparsely allocated.

Until now, the experiments use a schedule at 100% duty cycle and a single-hop network, see Table 1. All slots are either allocated for transmission or reception. Figure 5 shows the handshake duration when the network uses a more realistic duty cycle, and it is up to two hops deep. The MSF algorithm maintains the schedule. We choose a conservative DTLS timeout to prevent spurious retransmissions when lowering the duty cycle of the nodes. For both TLS and DTLS, we maximize the handshake message size, and we activate Nagle’s algorithm for TCP. For the single-hop setup, the latency of both the TLS and DTLS handshake is quite similar. Below a 25% duty cycle, the delay induced by the TCP acknowledgment packets becomes more significant. When we repeat the same experiment with a two-hop network, the behavior is similar and amplified.

### 3.3. Handshake Reliability

IEEE802.15.4 provides a reliable link and physical layer. Channel hopping and dedicated cells in the schedule mitigate many transmission errors due to multipath fading and collisions. The link layer uses acknowledgments, and OpenWSN performs up to 15 retransmissions combined with a back-off mechanism to minimize loss. However, constrained devices can drop IEEE802.15.4 frames due to limited packet buffer space on the constrained devices. To test how both TLS and DTLS behave when the lower layers do not provide 100% reliability, we set up an experiment where 6LoWPAN fragments have a 5% probability of being dropped when traversing the 6TiSCH network.

**Figure 6:**
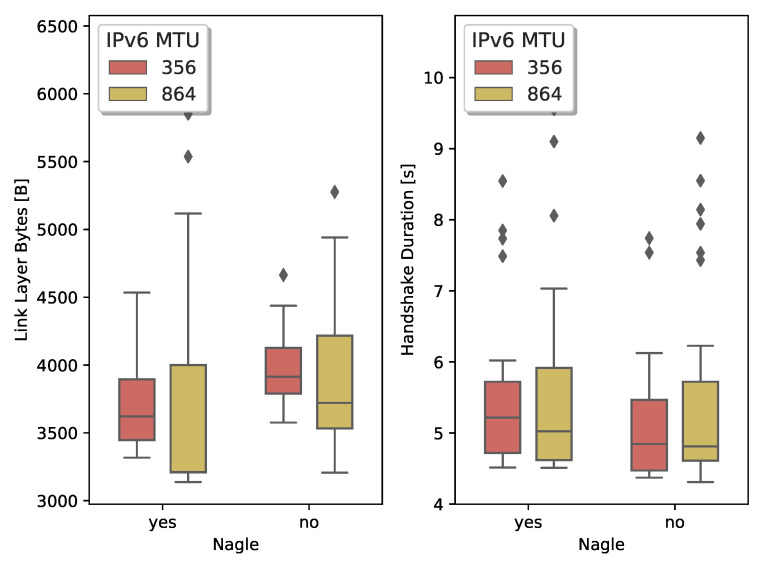
Limiting the MTU, by negotiating a small Maximum Segment Size (MSS) in the TCP handshake, reduces the number of bytes retransmitted and improves the handshake duration.

Figure 6 shows the results for the TLS handshake. We compare the lower whiskers (which correspond to a handshake with no losses) with the median. We notice, with Nagle active, an increase between ±10% and ±2% in the number of transmitted bytes for an MTU of 356 B and 864 B, respectively. Similarly, we see an increase between ±10% and ±15% when Nagle is not active. Comparing the handshake duration, we measure an increase between ±15% and ±10% with Nagle active and ±11% with Nagle inactive. We also notice that when TCP uses a larger MSS, the worst-case scenario deteriorates quickly.

We conclude that a lower MSS/MTU results in more bytes exchanged to establish the secure connection due to the header overhead. However, when there are losses, the small TCP segments allow to more precisely indicate, through the TCP SACK option, which segments require retransmission. When the receiver can accurately indicate which losses occurred, the transmitter can minimize the data it has to retransmit.

**Figure 7:**
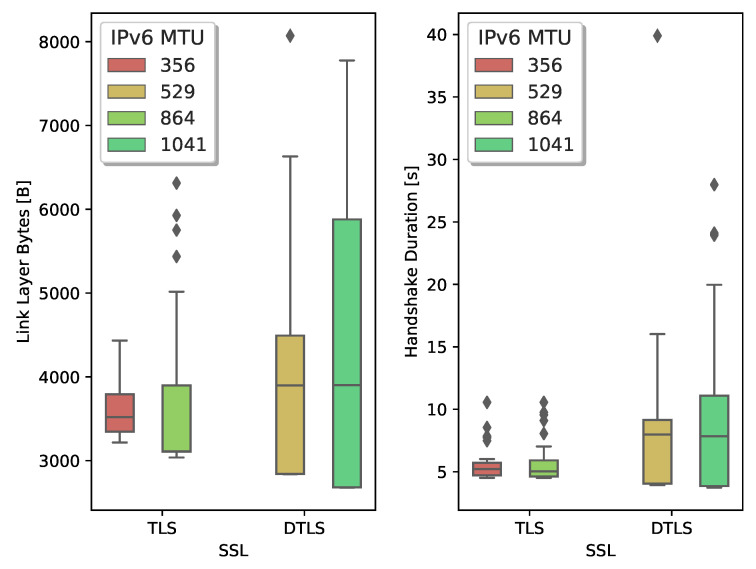
Loss of a 6LoWPAN fragment causes significant retransmission activity during the DTLS handshake. A properly configured TCP stack recovers the lost fragment with minimal impact on the 6TiSCH network.

In a second experiment, we look at the behavior of the DTLS handshake over lossy links and compare it to the values obtained for TLS. Figure 7 depicts the comparison between the lossy TLS and DTLS handshake. For an MTU of 529 B and 1041 B we measure an increase of ±31% and ±45%, respectively. Handshake duration roughly doubles.

The lack of control over the retransmission mechanism of DTLS results in a drastic increase in the number of bytes exchanged and total handshake duration. Since UDP combines many DTLS records of a flight in large datagrams, losing a single 6LoWPAN fragment results in the loss of all DTLS records. DTLS will then have to retransmit the entire flight.

## 4. Insights: The (D)TLS Handshake over 6TiSCH

### 4.1. Handshake Reliability

The results obtained in the previous section show that the retransmission behavior of DTLS is ill-suited for constrained networks. First, both endpoints must be capable of estimating network latency correctly to set an appropriate static value for the retransmission timeout. The default DTLS timeout of 1 s is too aggressive in most scenarios. Estimating network latency in a 6TiSCH network is tricky because it depends on the number of hops packets need to traverse, the density of the TSCH schedule (bandwidth), and the size of the packet. The 6TiSCH network fragments large IPv6 packets into multiple 6LoWPAN frames. The 6TiSCH devices send each fragment in an available active slot in the TSCH schedule. A network with a depth of multiple hops, combined with a sparse schedule, induces high latency on an end-to-end connection. The constrained endpoint in the 6TiSCH network should be aware of these factors impacting the RTT. An arbitrary DTLS endpoint on the Internet probably does not know that its messages will travel over a constrained 6TiSCH network.

**Figure 8:**
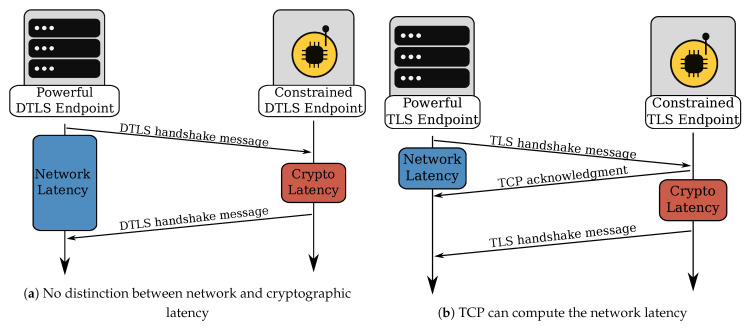
Computational-intensive computations can cause spurious retransmissions during the DTLS handshake. TCP’s acknowledgments indicate that the TCP segment arrived correctly and no retransmission is necessary, even if a reply is not immediately received.

Secondly, the cryptographic computations necessary to complete the handshake often take a long time on low-power devices, especially when they lack hardware acceleration. The RTT can significantly increase when constrained devices are computing the cryptographic functions, see Figure 8a. Unless the Internet DTLS endpoint has a very conservative initial retransmission value for the DTLS handshake, the network and cryptographic latency will cause many spurious retransmissions. Since DTLS retransmits entire flights—which can easily reach more than 1000 for flights four and five—this has dire consequences for the operation of the 6TiSCH network.

TLS relies on TCP to provide the necessary handshake reliability. TCP segments carry entire or partial TLS records, with each segment having its retransmission timer. Contrary to UDP, TCP uses acknowledgment packets in combination with its retransmission timers. The acknowledgments provide TCP with RTT estimation capability, see Figure 8b. The retransmission timer is initialized at 1 s upon completion of the TCP handshake and gets updated throughout the connection lifetime. TCP’s adaptive RTT estimation provides a significant advantage over the static timeout values of DTLS. However, the estimation happens on a per-segment basis and does not account for possible 6LoWPAN fragmentation. Smaller TCP segments can traverse the TSCH network quickly since they do not require 6LoWPAN fragmentation. Larger segments exhibit a much higher RTT due to 6LoWPAN fragmentation. When multiple small TCP segments are followed by a large segment, the Retransmission Timeout (RTO) value is too aggressive and causes a spurious retransmission of the large segment. We can limit the impact of spurious retransmissions by activating TCP’s SACK option [22]. The option is advantageous in a scenario where multiple TCP segments are on the wire, and segments successfully received are interleaved with segments lost. A receiver can then add one or more SACK blocks to its acknowledgment packet to precisely indicate which segments need retransmission.

### 4.2. Fragmentation

The maximum size of a (D)TLS record is 16,535 B. A (D)TLS record is the unit of protection, meaning that the encryption and Message Authentication Code (MAC) are calculated over an entire record. The record must be fully received before it can be processed. In theory, both (D)TLS endpoints require an output and input buffer of 16,535 B to store records. Typically, constrained devices do not have sufficient RAM for buffers of this size. Several approaches are available to ensure the size of the incoming and outgoing handshake messages is limited.

**Figure 9:**
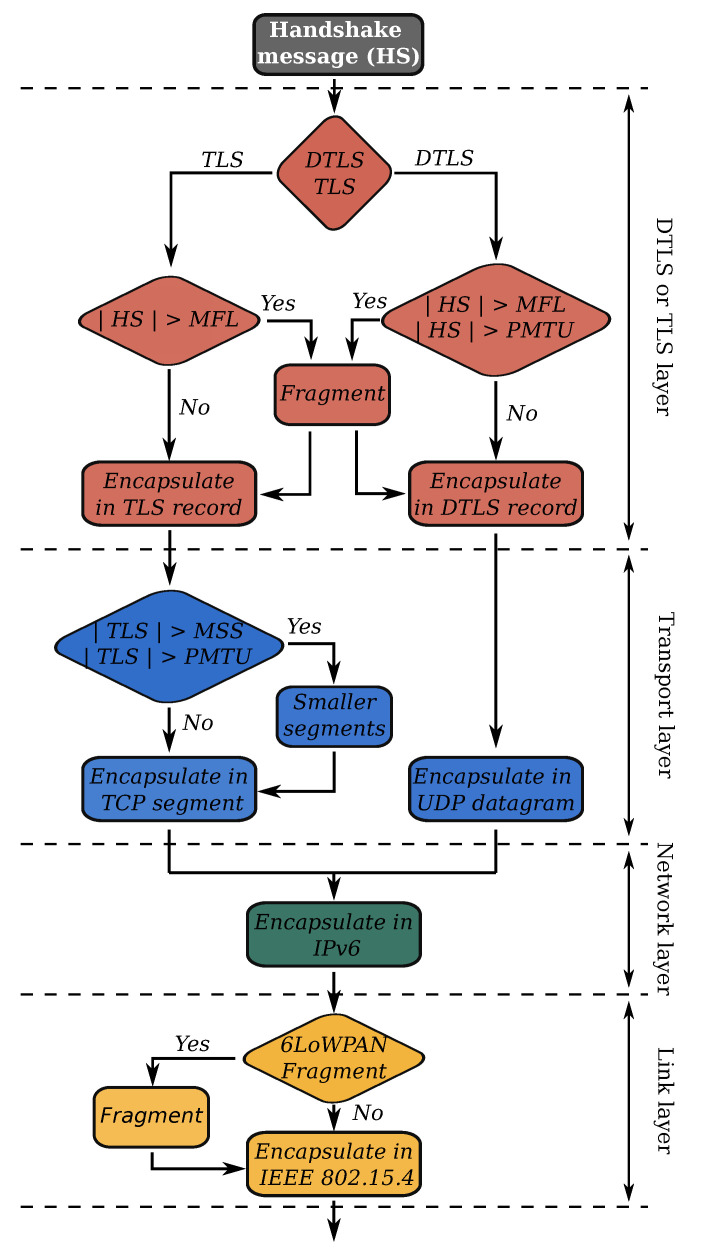
The different layers on which fragmentation occurs before a handshake message is sent by the IEEE802.15.4 radio.

By default, the DTLS handshake protocol supports the fragmentation of handshake messages to make sure that they do not exceed the PMTU, see Figure 3 and Figure 9. Large handshake messages can frequently occur when the messages contain certificate chains. In a 6TiSCH network, the PMTU depends on the number of 6LoWPAN fragments the individual constrained devices along the path can handle. In addition, the constrained destination must store all the fragments before reassembly. When the nodes are IPv6-compliant (MTU of 1280 ), they should at least be capable of storing 11 6LoWPAN frames.

To give the other endpoint an early warning about the limited PMTU, DTLS clients can use the MFL extension. When supported, MFL limits the size of the record fragments to 512 B, 1024 B, 2048 B or 4096 B. The main drawback is that MFL is optional and thus not supported by all DTLS endpoints. In addition, MFL negotiation can only be triggered by the DTLS client; the DTLS server cannot indicate to the client it wishes to limit the record fragment size. To address these issues the “record size limit” extension was defined. It is valid for all (D)TLS version and supposed to replace the deprecated MFL extension [23].

TLS endpoints do not have a built-in fragmentation mechanism to limit the size of the handshake messages, but TLS depends on TCP to appropriately size its segments Figure 9. A constrained device can make use of TCP’ Maximum Segment Size (MSS) option to explicitly set an upper bound to the segment size. In addition, TLS could use the MFL extension to reduce the record fragment size.

**Figure 10:**
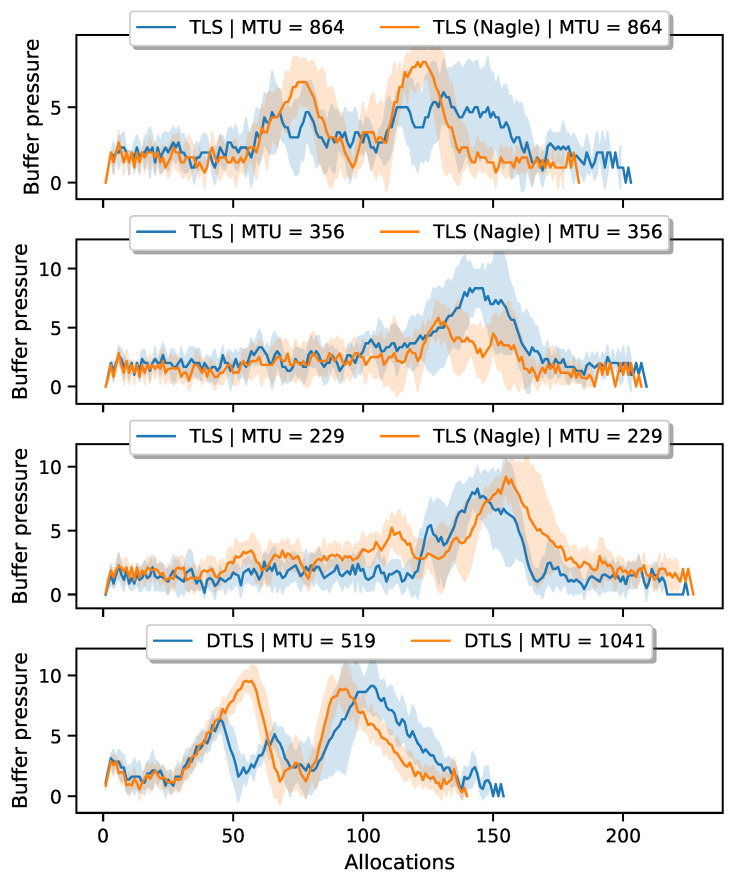
Measured buffer pressure on the constrained endpoint for different network configurations. The y-axis shows the number of packets in the queue. The x-axis shows the number of calls to the packet allocation function in OpenWSN.

To analyze the impact of different MFL and MSS sizes on the fragmentation load in the network, we set up an experiment that measures the 6LoWPAN buffer pressure on the constrained (D)TLS endpoint. We do not consider the buffer pressure of the intermediate router nodes since they use fast fragment forwarding. 6LoWPAN fragments are not reassembled on the routers and are forwarded directly to the next hop [24]. As long as intermediate routers have sufficient active slots—allocated by the SF algorithm—their buffers should not overflow. Figure 10 shows the results for varying configurations for both TLS and DTLS. We notice that for large handshake messages, Nagle’s algorithm increases the buffer pressure (see the top plot). Recall that Nagle will try to fill up a TCP segment before transmission. The larger TCP segments require many 6LoWPAN fragments. We can clearly distinguish two phases in the handshake protocol. The initial peak corresponds to the reception of a group a 6LoWPAN fragments containing the *ServerCertificate*, *ServerKeyExchange*, *CertificateVerify*, and *ServerDone* messages. The second bump is the accumulation of 6LoWPAN fragments being queued for transmission, after the node has prepared its *ClientCertificate*, *ClientKeyExchange*, *CertificateVerify*, *ChangeCipherSpec*, and *Finished* messages. Without Nagle, the endpoints are free to split the TLS record data over several smaller segments, resulting in fewer 6LoWPAN fragments per IPv6 datagram. It does increase the total amount of bytes, as shown in Table 2, due to more header overhead and additional TCP acknowledgments.

The behavior changes when we lower the handshake message size to 356 B. The initial peak, caused by the incoming server handshake messages, has completely disappeared. Since the segments are smaller, the receiving side needs to store fewer 6LoWPAN fragments before it can reassemble the original packet. Next, the node quickly prepares several short TCP segments (maximum size is 356 ), containing the *ClientCertificate*, *ClientKeyExchange*, *CertificateVerify*, *ChangeCipherSpec*, and *Finished* messages. When Nagle is not active, not all TCP segments are used at maximum capacity, which leads to more buffer spaces being occupied. With Nagle activated, fewer segments are created.

The DTLS handshake behaves similarly to a TLS connection that uses a large IPv6 MTU and Nagle’s algorithm. DTLS sends entire flights at once, automatically causing many 6LoWPAN fragments in transit. A constrained endpoint must correctly receive all fragments before reassembly can take place. Forcing a lower handshake message size with the MFL extension reduces the buffer pressure for incoming messages, but does nothing to alleviate buffer pressure for outgoing messages.

### 4.3. Burst Traffic

Section 2 shows how 6TiSCH uses its SF to manage the available bandwidth on the link-layer by allocating and deleting slots in the TSCH schedule. It is also the responsibility of the SF to ensure that limited packet buffers used by the constrained endpoint do not overflow, which would lead to packet loss. 6TiSCH defines a default MSF but allows developers to design their own SF. Of particular interest in our scenario is an SF that handles bursty network traffic. Domingo-Prieto et al. [25] proposed a fully distributed SF that manages the TSCH schedule through a Proportional, Integral, and Derivative (PID) control algorithm. The authors show that their approach obtains promising results in case of sudden traffic surges.

**Figure 11:**
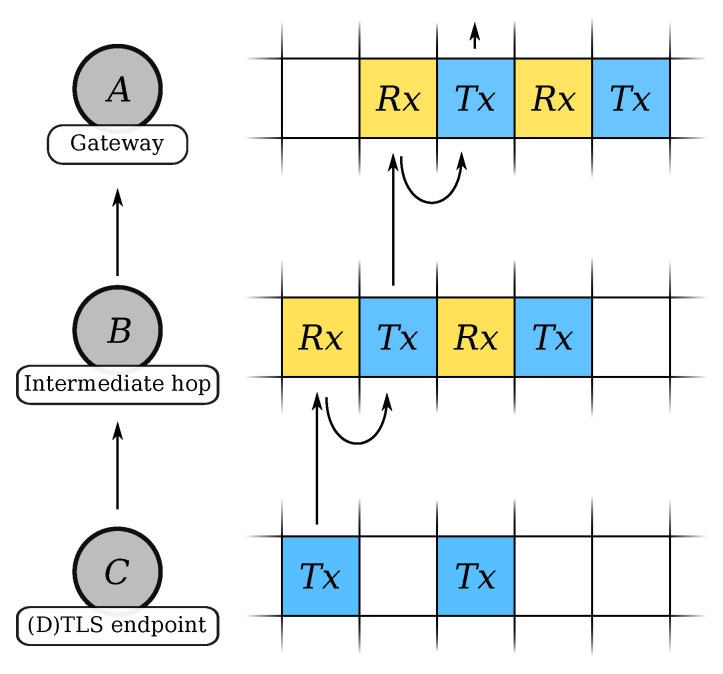
Slots are allocated in an interleaved, continuous manner to build a tunnel from the (D)TLS endpoint (C) over the intermediate hop (B) to the gateway (C).

Alternatively, we could extend the 6P commands to build a virtual tunnel in the TSCH schedule. The tunnel would allow a burst of IEEE802.15.4 frames to traverse multiple hops quickly to the gateway. All 6P commands follow the same generic structure, defined in [13], containing 2 B of opaque metadata. The SF and not by the 6P protocol interprets these 2 B. We could use the opaque metadata to instruct the SF to forward the slot allocation request recursively to its parent until it reaches the root, see Figure 11. Similarly, a slot deletion request can recursively clean up the previously allocated slots.

Besides the 6P protocol, a constrained device could use TCP’s traffic congestion algorithms. However, the traditional congestion mechanisms—Additive Increase/Multiplicate Decrease (AIMD) and TCP slow start—are not well-suited for wireless networks. The congestion mechanism increases or decreases the size of the congestion window, which is typically up to 4 times the MSS [26]. Alternatively, RFC6928 [27] defines an experimental new value for the initial congestion window, which in practice results in an initial window of 10 times the MSS. The latter is nowadays used in many TCP implementations [16]. In case of bursty traffic like the TLS handshake, an initial small congestion window could limit the sending rate if a low enough MSS was negotiated and rapidly allow for more data in flight. Nagle’s algorithm, see Algorithm 1 can also help limit congestion by maximizing the efficiency of the buffer space on the constrained devices.

The IETF draft on lightweight TCP [16] also mentions the use of the Explicit Congestion Control (ECN) bit in combination with TCP to limit network congestion. ECN allows a router (intermediate node) to signal a warning for looming congestion by setting a bit in the IP header of a packet, for example, when the internal buffer reaches 75% of its capacity. An ECN-enabled TCP receiver echoes back the congestion warning to the TCP sender by setting the ECN flag in its next acknowledgment. The sender then triggers congestion control measures as if a packet loss had occurred.

Finally, the use of delayed acknowledgment packets can also help reduce congestion. TCP’s delayed acknowledgments are meant to reduce the number of acknowledgment packets sent within a TCP connection, thereby reducing network overhead. However, it is well-known that delayed acknowledgments should not be used in combination with Nagle’s algorithm since this would impact network throughput.

Congestion control is not available when using UDP, and DTLS does not incorporate any of the TCP congestion control mechanisms. DTLS can, however, still use the 6P tunnel-building mechanism described above. Alternatively, the Datagram Congestion Control Protocol (DCCP) [28] can carry DTLS messages. It provides congestion control for unreliable datagrams.

## 5. Performance Enhancing Proxy for TLS/TCP

Even though TCP’s adaptive retransmission algorithm is a clear improvement over the static timers used in DTLS (see Figure 7) it is still oblivious of the fragmentation occurring at the 6LoWPAN layer and the additional RTT this incurs. The latter can cause spurious retransmissions which are particularly expensive in constrained networks. In the experiments presented in Section 3, we solve this issue by dropping the initial *synack* segment during the TCP handshake. It causes a retransmission of the *synack* segment but opens the connection with a more conservative fallback RTO value of 3 s instead of 1 s [29]. It ensures that TCP does not start with round-trip time (RTT) estimation that is too aggressive, triggering needless retransmits during the TLS handshake. It is not possible to easily change the initial TCP RTO from user space. Updating this value in the code would require the recompilation of the Linux kernel.

To solve the problem in a more elegantly, we use a TCP Performance Enhancing Proxy (PEP) [11]. PEPs are a commonly used technique to accelerate TCP connections over satellite links without tampering with the TCP implementation details. It operates as follows:

1. The PEP intercepts a TCP connection before the segments are sent to the satellite, and it terminates the connection as if the interceptor is the intended destination. It immediately sends an acknowledgment back to the original sender.
2. It forwards the TCP segments further to the actual destination, but it accounts for the specifics of the satellite link, notably a long RTT.
3. In case of segment loss, the PEP takes care of retransmissions.

For the original TCP endpoints, the TCP PEP remains transparent. In a 6TiSCH network, the PEP can be cohosted with the network gateway (root). The network gateway intercepts the TCP connection and quickly generates acknowledgments for segments originating from the Internet, thereby preventing retransmissions due to fragmentation-induced latency. Since the network gateway is aware of the specifics of the 6TiSCH network, it can calculate a novel RTT, which takes into account the fragmentation rate and the allocated bandwidth in the 6TiSCH network. We implement the TCP PEP in OpenVisualizer and assess its performance, see Table 3.

We notice that the TLS handshake takes one second less to complete compared to Table 2. Since the PEP acknowledges the segments coming from the server, we no longer need to drop the initial *synack* to force TCP to use a more conservative initial RTO. Surprisingly, the TLS handshake even completes faster than the DTLS handshake, even though it exchanges more bytes in total. However, the initial DoS protection of DTLS immediately triggers 6LoWPAN fragmentation and adds reassembly delays, while the TCP handshake packets are small and quickly traverse the 6TiSCH network. DTLS’ MTU is also larger— 1041 B vs. 864 B—which again causes more delays during fragment reassembly. The TCP acknowledgments are small and do not require fragmentation. It limits their impact on the overall duration of the handshake. We can conclude that DTLS spends a significant amount of time waiting for all the 6LoWPAN fragments because of the high fragmentation rate of the UDP datagrams.

## 6. Improvements in (D)TLSv1.3

In 2013, the IETF started working on a new version of the TLS protocol, called TLSv1.3 [30]. TLSv1.3 entered RFC status in March 2018. The latest iteration of the TLS protocol contains some significant improvements that are also interesting for low-power devices. Not only does the new specification improve the security of the protocol but it also reduces the latency of the handshake phase. It now takes one full RTT less to complete. While TLSv1.2 takes two full RTTs until application data can be exchanged, TLSv1.3 by default only requires one RTT. The client tries to guess the key exchange algorithm the server is going to pick, allowing the client to send its key share during the first RTT. In case the client picks an unsupported algorithm, the server requests a new key share. Since the number of possible key exchange algorithms is drastically reduced in TLSv1.3 compared to TLSv1.2, there is a good chance the client chooses a supported algorithm.

At the time of writing, the DTLSv1.3 specification [31] is still in draft status at the IETF. In addition to the changes it inherits from the TLSv1.3 specification—faster handshake completion—it also updates some features specific to DTLS. It omits superfluous version numbers and type fields in the headers, it has a novel variable-length record header with support for a connection identifier and uses fewer bits to encode sequence and epoch numbers. However, the most important change concerning constrained devices is the introduction of a new content type called ACK. A DTLSv1.3 endpoint can use acknowledgment messages when it detects disruptions during the reception of a DTLS flight. The draft proposes to arm a timer for 1/4 of the duration of the current retransmission timer timeout after a disruption is detected. When the timer expires, the endpoint generates an acknowledgment for the parts of the flight that were well-received and correctly processed by including a list of the record numbers in the acknowledgment message. Upon reception of an acknowledgment the sender disables the retransmission timer and retransmits only the record fragments that were lost.

## 7. Related Work

The literature contains several works that study the performance of TLS and DTLS in the context of the IoT. They majorly focus the cryptographic overhead of the protocols and propose alternative schemes to offload the resource-demanding computations, in particular asymmetric cryptography [32,33,34,35].

The work presented by Vučinić et al. [36] considers the performance of transport layer security in constrained environments. The authors focus only on the DTLS protocol, but they investigate its performance in combination with two duty-cycled MAC-layer protocols: X-MAC, a preamble sampling protocol [37] and beacon-enabled IEEE 802.15.4 [38]. The results on handshake duration in multihop, duty-cycled networks, obtained through the Contiki Cooja simulator [39], are similar to ours. Our work extends the previous by considering the TLS standard as well, using the constrained IPv6-enabled 6TiSCH stack, implementing the experiments on state-of-the-art hardware, and presenting the side-by-side performance comparison with DTLS in different scenarios.

RFC7925 [40] describes generic DTLS and TLS profiles for constrained IoT devices. It does not alter the protocols but recommends specific configuration options to make the protocols reasonably implementable on most devices. Some of the recommendations made are:

- Set the initial timeout for the DTLS handshake to 9 s.
- Mandatory client support for the MFL extension.
- Session resumption (which requires less messages to be exchanged and only symmetric cryptography). In addition, when the server is constrained instead of the client, support for client-side handshake state storage [41].
- Use TLS-FALSESTART [42] to shave off one RTT and speed up the handshake duration.
- Optionally, use of the (D)TLS heartbeat extension [43] to verify whether the peer is still alive and keep the connection up (preventing a new handshake).

The first two recommendations are straightforward. Since the original default timeout value of 1 s is too aggressive for most constrained networks, a more conservative choice is proposed. The RFC argues that 9 s is big enough to absorb large latency variance due to slow computations or intrinsic network characteristics [40]. The mandatory client-side MFL support allows notifying the server of limited buffer space on the client. However, the server can ignore the extension. The remaining recommendations focus on preventing a new full handshake by either keeping the connection alive or using restricted versions of the handshake protocol. The TLS-FALSESTART and heartbeat extension necessitate additional code to the stack, while session resumption forces either the server or client to store state information.

## 8. Conclusions

In this article, we investigate the network performance of the TLSv1.2 and DTLSv1.2 handshake when messages are transferred over a 6TiSCH network. Although DTLS has been the defacto security protocol for the IoT in the past years, we show that some caution is advised when using it to protect application traffic in low-bandwidth, multihop networks. The unreliability of the underlying transport layer forced the DTLS designers to reimplement several TCP-like features in the DTLS handshake protocol. However, these mechanisms are less flexible than their TCP counterpart. On the one hand, DTLS’ handshake outperforms TLS’ handshake when it has an ideal communication link between both peers. It uses between 500 B and 800 B less, and completes roughly 0.6 s faster. On the other hand, the performance of the DTLS handshake degrades rapidly when the link quality deteriorates, the performance of the TLS handshake remains more stable in the same conditions. When transport layer packets have a 5% probability of getting lost, the DTLS handshake duration more than doubles, and it uses between ±37% and ±45% more bytes. The TLS handshake duration grows between ±10% and ±15%, and the number of exchanged bytes grows between ±2% and ±15%. The different TCP options allow a developer to tailor the TCP stack and the TLS handshake to the characteristics of the constrained network.

Besides the handshake comparison, we also propose an improvement for the TLS handshake based on a TCP PEP. The PEP aims to resolve the issues that arise when 6LoWPAN fragmentation adds network latency, hidden to the TCP retransmission mechanism. Experimental results show that the PEP accelerates the handshake and removes any spurious retransmissions.

Our current work focuses on analyzing the performance of TLSv1.3 and DTLSv1.3 in constrained networks.

## Figures and Tables

**Table 1:** 6TiSCH and (D)TLS configuration.

Parameter	Value
Cipher suite	tls_ecdhe_ecdsa_with_aes_128_ccm_sha256
Curve	SECP192R1
DTLS timeout	3 s
Authentication	Mutual (single certificate in chain)
Scheduling func.	Minimal Scheduling Function (MSF)
Slotframe length	5
Duty cycle	100%
Cells	1 Shared TxRx, 1 autonomous Rx, 3 managed cells
Topology	Single hop
TCP options	MSS, Nagle, SACK, and Delayed ACKs
UDP header size	Compressed to 4 B with 6LoWPAN

**Table 2:** Impact of the Maximum Transmission Unit (MTU) on the (D)TLS handshake.

SSL	Negotiated [B]		MTU [B]	Latency [s]		Bytes [B]		Nagle
	MSS	MFL		μ	σ	μ	σ	
TLS	844	-	864	4.495	0.096	3102	5.49	✓
	844	-	864	4.285	0.076	3358	0.60	-
	336	-	356	4.539	0.089	3345	1.25	✓
	336	-	356	4.241	0.118	3671	3.74	-
DTLS	-	1024	1041	3.795	0.064	2680	1.00	-
	-	512	529	3.891	0.090	2841	0.83	-

**Table 3:** Performance-enhancing proxy and TLS.

SSL	Negotiated	MTU [B]	Latency [s]		Bytes [B]		Nagle
	MSS		μ	σ	μ	σ	
TLS	844	864	3.323	0.125	3098	2.13	✓
	844	864	3.209	0.083	3359	1.15	-

## Data Availability

The source code used for the experiments is hosted at https://github.com/openwsn-berkeley/openwsn-fw and https://github.com/ARMmbed/mbedtls (accessed on 1 February 2021).
